# Rich-club in the brain’s macrostructure: Insights from graph theoretical analysis

**DOI:** 10.1016/j.csbj.2020.06.039

**Published:** 2020-06-29

**Authors:** Dae-Jin Kim, Byoung-Kyong Min

**Affiliations:** a Department of Psychological and Brain Sciences, Indiana University, Bloomington, IN 47405, USA; b Department of Brain and Cognitive Engineering, Korea University, Seoul 02841, Republic of Korea

**Keywords:** MRI, magnetic resonance imaging, DTI, diffusion tensor imaging, EEG, electroencephalography, MEG, magnetoencephalography, MDD, major depressive disorder, ASD, autism spectrum disorder, ADHD, attention deficit hyperactivity disorder, BD, bipolar disorder, AD, Alzheimer’s disease, TBI, traumatic brain injury, Rich-club, Brain connectivity, Brain network, Graph theory, Neuroimaging

## Abstract

The brain is a complex network. Growing evidence supports the critical roles of a set of brain regions within the brain network, known as the brain’s cores or hubs. These regions require high energy cost but possess highly efficient neural information transfer in the brain’s network and are termed the rich-club. The rich-club of the brain network is essential as it directly regulates functional integration across multiple segregated regions and helps to optimize cognitive processes. Here, we review the recent advances in rich-club organization to address the fundamental roles of the rich-club in the brain and discuss how these core brain regions affect brain development and disorders. We describe the concepts of the rich-club behind network construction in the brain using graph theoretical analysis. We also highlight novel insights based on animal studies related to the rich-club and illustrate how human studies using neuroimaging techniques for brain development and psychiatric/neurological disorders may be relevant to the rich-club phenomenon in the brain network.

## 1 Introduction

A network represents a formal mathematical model in which a complex system can be decomposed into elements (i.e., nodes or vertices) and their interactions (i.e., edges, links, or connections). The comprehensive structural description of the brain as a network of neural elements and their interconnections is known as the connectome [1]. Structural scales in the nervous system range from molecules to the whole brain [2], and the association between these elements in the brain network is generally described by its structural (or functional) connections at four different scales: *macroscale* at the level of gray matter, *mesoscale* at the level of neuronal subgroups, *microscale* at the level of individual neurons, and *nanoscale* at the level of synapses [3]. From a network perspective, brain functions are considered to be highly dependent on the brain’s structural network architecture at each scale [4]. Accordingly, several fields in modern network neuroscience utilize their own approach to studying brain connections depending on the acquired level of the dataset (i.e., elements). Recent developments in noninvasive techniques for mapping brain connectivity enable better characterization of the structural and functional properties of a specific neuronal system [139].

**Fig. 1:**
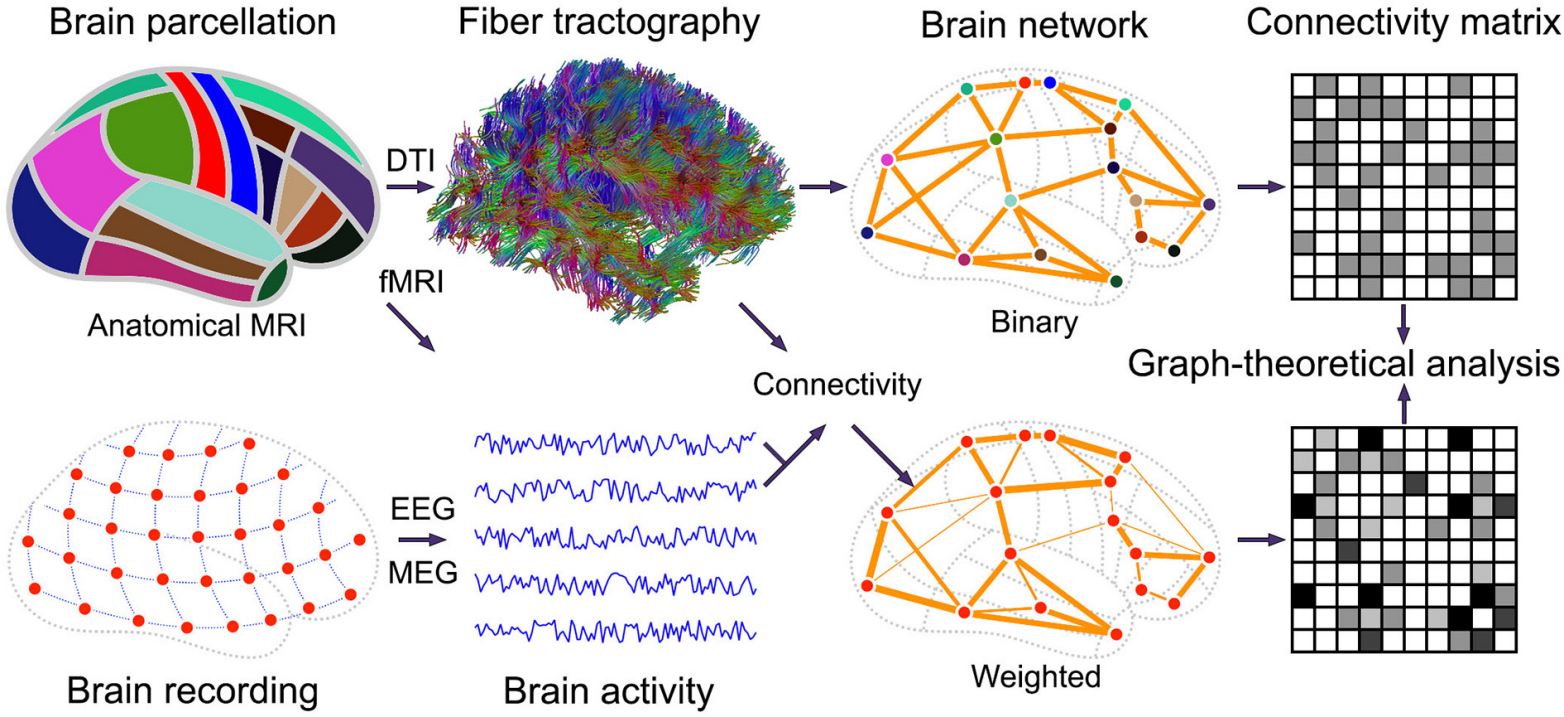
The construction of brain networks using neuroimaging and neurophysiological data. For a structural network, the brain is parcellated into a number of segregated regions, often using high-resolution anatomical magnetic resonance imaging (MRI). Subsequently, the fiber tracts are generated using diffusion tensor imaging. Structural connectivity may indicate the existence or weight of a connection between two parcellated regions, resulting in a connectivity matrix. For a functional network, the time-varying brain activity measured with electrophysiological signal measurements or functional MRI can be used to compute functional interdependence leading to functional connectivity. Graph theoretical measures (Table 1) can be computed using the structural and functional connectivity matrix.

**Fig. 2:**
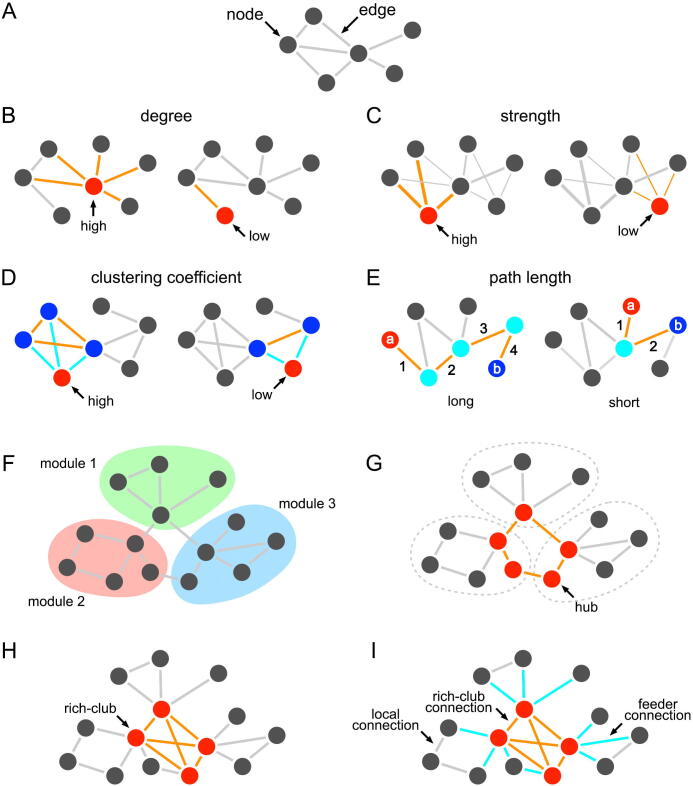
Basic graph theoretical measures. (A) Networks are described as a set of nodes and edges, in which nodes can be a set of neurons, brain regions, or specific recording sites, and edges can be either structural connections or functional relationships between two nodes. (B) Degree is defined by the number of connections of each node. (C) In a weighted network, the sum of the weights connected to the node represents the strength. (D) The highly clustered node (red) has three neighboring nodes (blue), and they are also connected (orange) to form a cluster. (E) Path length represents the sum of steps (or connection distances) required to travel from one node to another. For example, four steps are required from node *a* (red) to *b* (blue) for the left network, while two steps are required for the right network. (F) Highly clustered nodes are likely to have more mutual connections to form a module. (G) Some nodes in a network may play central roles as hubs for network information transfer. (H) Rich-club nodes represent a set of hub nodes that are also highly connected with each other. (I) Rich-club, feeder, and local connections represent the connections only among rich-club nodes, between rich-club and non-rich-club nodes, and only among non-rich-club nodes, respectively. (For interpretation of the references to colour in this figure legend, the reader is referred to the web version of this article.)

The structure of networks has been analyzed within a mathematical framework known as *graph theory* (Fig. 1). Using graph theory, networks including neural systems such as the brain can be described as a quantitative and comparative model of real-world systems at all scales (i.e., macro, meso, micro, and nano) and modalities (e.g., single-cell recording, neuronal tracing, and neuroimaging) [5]. An early finding from the macroscopic brain network perspective was that the human brain is organized in a highly efficient manner for integrated information transfer [6], known as *small-world topolog*y, as anticipated in several biological, technical, and social networks [7]. Two assumptions are postulated to form a small-world network. First, a subgroup of network elements should form dense, interconnected clusters to confirm local network segregation, as defined by a *clustering coefficient* [7]. A higher clustering coefficient of each network element often leads to network communities or modules (Fig. 2 and Table 1). Second, lengths or distances between any pairs of network elements, often defined by the reciprocal of the connectivity strength, should be shorter for a greater degree of global integration, resulting in a lower *shortest path length* [7]. The small-world topology designates networks in which the clustering coefficient is significantly larger than (and the shortest path length is similar to) those of randomly connected networks, defined as *small-worldness* [8]. However, the existence of small-world topology provides limited information on network architecture and has several pitfalls in terms of its evaluation, utility, and interpretation [5], [9]. As such, more appropriate network measures such as *modularity* have been proposed to characterize local and global network architecture [10]. Network modules (i.e., communities or clusters) are defined by a set of network elements with a number of interconnections within each module and fewer connections among modules [11], [12].

**Table 1:** Description of graph theoretical measures.

Measure	Description
Graph	A set of nodes and edges (often called a network).
Node	In a brain network, a node can be a single neuron, set of neurons, brain region, or specific recording site (e.g., electroencephalography electrode position). Each node has a unique topological location within a network.
Edge	An edge, also called a connection or link, is defined by an interrelationship between two nodes. It may be structural or functional. Simply, it could indicate the existence of a connection (i.e., binary, 0 or 1) or strength (i.e., weight; e.g., neural density or functional correspondence). It may have a direction (e.g., positive to the efferent connection and negative to the afferent connection).
Neighborhood	Connected nodes to a node by an edge forming a subnetwork.
Degree	The number of edges attached to a node.
Strength	Sum of the edge weights attached to a node, when all network edges have their own weights (i.e., weighted network).
Clustering coefficient	The probability that the neighborhood (i.e., connected) nodes for a node are also connected to each other. The *global* clustering coefficient for a network is computed by averaging clustering coefficients across all nodes in the network.
Distance	Topological length, often defined by an inverse of the edge weight (i.e., if the weight = 0, then the distance → ∞).
Shortest path length	Distance between two nodes. It may be the number of steps for a binary network or the sum of connection distances for a weighted network along the shortest path from one node to another.
Characteristic path length	Average shortest path length across all nodes in the network.
Small-worldness	Ratio of clustering coefficient and characteristic path length, which are normalized relative to those of the random networks. A small-world network is more clustered with a similar characteristic path length than degree-preserved random networks.
Efficiency	Average of the “inverse” of connection distances from a node to all other nodes refers to *nodal* efficiency. *Global* efficiency represents average of the nodal efficiencies of all nodes. *Local* efficiency is computed at a node and its neighborhood subnetworks. Contrary to the path length, efficiency is less influenced by isolated nodes (i.e., if the path length → ∞, then the efficiency = 0).
Module	A set of highly connected nodes. In general, the number (or strength) of connections within modules is more than that between modules.
Hub	Nodes with topologically important roles in a network. A hub can be a node with high degree or strength (often called a core).
Rich-club	A set of hub nodes with more connections to each other. A subnetwork with only rich-club nodes should have more connections than a random network with the same degree and edge distributions.

A macroscopic structural brain network derived from anatomical magnetic resonance imaging (MRI) has revealed groups of cortical regions that are morphologically connected, subserving distinct brain functions such as language, motor, and visual functions [13]. More detailed analyses using diffusion spectrum imaging and higher resolution of cortical parcellation have identified six structurally distinct modules comprising posterior medial and parietal cerebral cortices, and several distinct temporal and frontal modules [14]. Several findings have suggested the following: (1) brain network modules possess a large number of relatively short connections among adjacent brain regions, and (2) these modules are interconnected via a limited set of network nodes termed *network hubs,* which play central roles in neuronal information flow. Hub regions in brain network modules are defined by network nodes with a larger number of connections and have been identified in the cat, macaque [15], and human brain [14]. Hubs are classified into two categories: *connector hubs* corresponding to the interconnection among network modules and *provincial hubs* within each network module. Damage to connector hub regions (e.g., lesions) causes larger disturbances across widespread brain networks [16], [17].

The concept of the *rich-club* phenomenon within a network arose due to the observation that certain hub nodes of a network are more densely interconnected among themselves than other non-hub nodes [18]. Evidence for the rich-club phenomenon in other biological networks such as the protein interaction network is insufficient, suggesting a higher level of functional specification solely of densely connected network nodes. In this regard, several networks including scientific collaboration networks [18] and power grids [19] exhibit rich-club properties, implying a critical role of rich-club nodes for global communication in the most efficient way. In the human brain network, the existence of rich-club organization has been detected, and certain hub regions are considered rich-club members which form tight subnetworks by themselves [20]. Following the initial discovery of rich-club organization in macroscopic structural networks of the human brain, several studies have demonstrated the characteristics of the rich-club phenomenon in terms of cost-efficiency theory, and brain development and diseases [21], [22], [23], [24], [25].

In the present review, we examine the key contribution of rich-club approaches to understanding its potential roles in brain networks. We first briefly summarize the mathematical background underpinning key concepts of rich-club topology. We then clarify explicit findings in *Caenorhabditis elegans*, the only organism for which the complete neuronal wiring diagram has been mapped. We also assess relevant rich-club data for mammals such as mice, rats, and cats. We then review the background and recent advancements in network studies focusing on the rich-club and structural neuroimaging in human brain development and psychiatric/neurological applications, which constitute one of the most extensively researched areas of the rich-club. We conclude this review by highlighting future prospects in relation to the effectiveness and potential use of this computational and theoretical tool.

## 2 Construction of neuronal networks

Serial reconstruction of electron micrographs was initially used to elucidate the complete connectome, particularly for *Caenorhabditis elegans* [26]. This worm has a relatively small number of neurons, which enabled manual reconstruction of its complete neuronal wiring diagram. Nevertheless, this approach suffered from its labor-intensive nature, in particular for neural systems with a larger number of neurons such as the mammalian brain. As a suitable alternative to investigate macroscale neural networks, MRI has been used extensively to define whole brain wiring diagrams *in vivo* for larger neural systems, enabling the comparison of network characteristics across species. Since the introduction of white matter fiber tractography, an MRI-based 3D reconstruction technique that visualizes neural tracts using water diffusivity in the brain predominantly with diffusion tensor imaging (DTI) [27], noninvasive neuroimaging methods such as DTI have been utilized for macroscopic brain network analysis. Other methods for constructing complete whole-brain networks include functional modalities such as functional MRI (fMRI), electroencephalography (EEG), or magnetoencephalography (MEG), by computing the statistical interdependency as a measure of the network edge between spatial locations. Of these, structural neuroimaging modalities such as DTI have overarching benefits from several aspects [28], [29]. First, the DTI technique is specialized for extracting white matter connections and is suitable for macroscale network analysis to investigate larger functional and structural brain subregions (e.g., Brodmann areas). Second, it can reduce computational complexity, which often arises due to the large number of network elements and interactions. For example, the human brain comprises approximately 16 × 10^9^ neurons in the cerebral cortices and 69 × 10^9^ neurons in the cerebellum, with more than 10^12^ neuronal connections [30], [31]. DTI has a 1–2-millimeter resolution, comprising millions of neurons in an acquired voxel, which enables whole-brain network analysis with a reasonable number of network constituents (e.g., 52 distinct Brodmann regions with distinct anatomical parcellations). Third, when combined with functional neuroimaging techniques (e.g., resting and task fMRI), an intuitive approach for examining structural and functional associations is afforded [4], [32].

## 3 Detection of the rich-club

Zhou and Mondragon [33] defined rich-club elements as a set of highly interconnected nodes forming a tight subnetwork within a network. The mathematical description of the rich-club^1^ phenomenon is provided by the rich-club coefficient *ϕ* [18] as follows:

$$\phi \left(k\right)=\frac{2E_{>k}}{N_{>k}\left(N_{>k}-1\right)}$$

where *k* represents the number of connections attached to each network node (the *degree*); *N_>k_* represents the number of nodes whose degree is larger than a given value *k*; and *E_>k_* represents the number of connections of a subnetwork comprising *N_>k_* nodes. It should be noted that the rich-club coefficient *ϕ* is a function of the degree *k,* because it corresponds to the measure of the density of the subnetwork comprising nodes with degree greater than *k*. Practically, the detection procedure involves the following steps: (1) for an *N* × *N* network matrix where *N* is the number of all network nodes, the degree (*k*) is computed at each node; (2) for each *k* (where 1 ≤ *k* < *N*), all nodes with degrees less than or equal to *k* are removed to construct a new *N_>k_* × *N_>k_* subnetwork; (3) the number of existing connections and possible maximum connections of the subnetwork are denoted by *E_>k_* and calculated by *N*_≥_*_k_*(*N*_≥_*_k_* − 1)/2, respectively; and (4) the rich-club coefficient *ϕ*(*k*) is computed as the ratio of *E_>k_* and *N*_≥_*_k_*(*N*_≥_*_k_* − 1)/2. Higher degree nodes in a randomly connected network (e.g., the Erdos-Renyi network, a random network with a Poisson degree distribution) tend to have a higher probability of being interconnected to each other by chance [18], [20]. Therefore, to evaluate the statistical significance of *ϕ*(*k*), the coefficient is typically normalized to the rich-club coefficient *ϕ_random_*(*k*) computed from a set of random uncorrelated networks with preserved degree distribution:

$$\phi_{\mathrm{normalized}}\left(k\right)=\frac{\phi \left(k\right)}{\langle \phi_{\mathrm{random}}\left(k\right)\rangle }$$

where 〈〉 represents the average. If the rich-club coefficient *ϕ*(*k*) of a network is larger than the average rich-club coefficient across randomized networks (i.e., *ϕ*(*k*) > $\langle \phi_{\mathrm{random}}\left(k\right)\rangle$ or *ϕ_normalized_*(*k*) > 1), the density of the subnetwork for *k* is considered to be higher than that of its randomized networks, and the network is considered to have a rich-club architecture. For a weighted network, the weighted rich-club coefficient *ϕ^w^* is calculated using the following equation:

$$\phi^{w}\left(k\right)=\frac{W_{>k}}{\sum_{l=1}^{E_{>k}}w_{l}^{\mathrm{rank}}}$$

where *W_>__k_* represents the sum of weights in the *N_>k_* × *N_>k_* subnetwork, $w_{l}^{\mathrm{rank}}\geq w_{l+1}^{\mathrm{rank}}$ with 1 ≤ *l* ≤ *E* represents the ranked weights of the links of the network [34], and the coefficient *ϕ^w^* should be normalized over a set of random networks. A unifying framework for the weighted network has been proposed by Alstott and colleagues [35]. In addition to using a network structural attribute (e.g., network degree) to compute rich-club phenomenon, Cinelli [36] recently suggested a generalized rich-club framework using non-structural information (e.g., social or technical attributes related to network nodes). In his work, instead of using only the network degree, any structural measures distinct to degree (e.g., node centrality measures) could also be used to evaluate rich-club ordering. Furthermore, when network nodes have a certain attribute which is not directly derived from the network structure itself (i.e., node metadata such as the wealth of each person in a social network), he suggested two types of network randomization:

$$\;\;\;\phi_{\mathrm{normalized}}^{\mathrm{rewiring}}\left(m\right)=\frac{\phi \left(m\right)}{\langle \phi_{\mathrm{random}}^{\mathrm{rewiring}}\left(m\right)\rangle }\;\;\;\;\phi_{\mathrm{normalized}}^{\mathrm{reshuffling}}\left(m\right)=\frac{\phi \left(m\right)}{\langle \phi_{\mathrm{random}}^{\mathrm{rereshuffling}}\left(m\right)\rangle }$$

where the value *m* corresponds to the value of the node metadata, and $\phi_{\mathrm{random}}^{\mathrm{rewiring}}$and $\phi_{\mathrm{random}}^{\mathrm{reshuffling}}$ represent rich-club coefficients with degree-preserving rewiring and metadata reshuffling, respectively. This generalization may be useful for investigating the importance of node metadata in a network and the association between topological and nontopological properties.

## 4 Rich-club of *Caenorhabditis elegans*

In 2013, Towlson and colleagues investigated the neural network of the nematode worm *Caenorhabditis elegans*, anatomically defined at a cellular scale with 2287 synaptic connections of 279 neurons [37], [38]. Towlson and colleagues determined 11 neurons as rich-club members, in which eight neurons (AVAR/L, AVBR/L, AVDR/L, and AVER/L) were located in the lateral ganglia of the head and three neurons in the lumbar (PVCR/L) and dorsorectal (DVA) ganglia. The efficiency of the subnetwork with only 11 rich-club neurons (i.e., *network efficiency* [39]) was 0.92, comparable to the efficiency of 268 non-rich neurons (i.e., 0.38). Most of the rich-club neurons (i.e., 10 of 11 rich-club neurons) are command interneurons with functional roles in forward or backward locomotion circuits [37], while DVA is a proprioceptive interneuron which modulates sensorimotor integration during locomotion [40]. Notably, 4% (~11/297) of these elite neurons were involved in 48% of the total connection distance and 52% of inter-modular connections, suggesting a critical role of rich-club neurons in communication between distant network modules. Towlson and colleagues also reported that rich-club neurons were generated in earlier developmental stages before the main phase of developmental elongation of the body, findings that were replicated by Ma and Mondragon [41]. The importance of the small number of modulatory neurons was revealed in the full hermaphrodite *C. elegans* network with all 302 neurons using the aminergic signaling map [42], in which the monoamine network contains a distinct rich-club comprising dopamine, serotonin, and tyramine-releasing neurons corresponding to sensory and motor activities. These findings were distinct to those of Towlson and colleagues [37] (i.e., interneurons) but further suggested a distinct functional rich-club phenomenon of extrasynaptic networks from synaptic networks in the same nematode worm. More recently, distinctive transcriptional properties of rich-club neurons in *C. elegans* alongside coupled gene expression have been reported, in which rich-club neurons exhibit similar gene expression regulating higher-order behaviors (e.g., locomotion by command interneurons) [43]. Thus, rich-club analyses using neural, chemical, and genetic transcription of this nematode worm may support investigations of higher-order functions and species-conserved mechanisms in the behavioral repertoire of other animals.

The rich-club studies on *C. elegans* are valuable as they contribute to deriving fundamental hypotheses on biological network formation. For example, an important aspect of network formation revealed by studies on *C. elegans* is the minimization of network costs (i.e., biological networks are likely to have less connections with shorter paths to minimize the spending of neural resources). However, to reduce costs in a “global” network, revised connection strategies may be more beneficial to facilitate more efficient information propagation. A selected set of network nodes (e.g., rich-club) enables the network to rewire certain connections with increased topological paths and to consequently reduce overall connection costs. Rich-club neurons in *C. elegans* are predominantly command interneurons related to locomotion, suggesting that the existence of rich-club members in a network is crucial to optimize the most important network function (e.g., movement in the case of *C. elegans*).

## 5 Rich-club of mammals

### 5.1 Cat

The cerebral cortex of mammals such as cats has a functionally subdivided modular structure (i.e., four main modules of modally-related areas with visual, auditory, somatosensory-motor, and fronto-limbic modules) [44], [45]. Gómez-Gardeñes and colleagues suggested a new module with highly connected but not necessarily module-related areas forming a rich-club connectivity pattern in the cat, in which the rich-club regions of the cat’s cerebral cortex (i.e., 53 network nodes + 826 cortico-cortical neural projections) [46] consist of 11 cortical areas (three visual areas: 20a, 7, and anterior ectosylvian sulcus; one auditory area: posterior part of posterior ectosylvian gyrus; two somatosensory-motor areas: medial area 6 and lateral area 5A; and five fronto-limbic areas: agranular and granular insula, posterior cingulate cortex, area 35, and area 36) [47]. Their findings emphasized that these rich-club areas enable a network transition in terms of its dynamics from a simple modular structure to global synchronization, related to higher cognitive tasks in mammals such as planning and integration. The impact of these rich-club regions has been investigated by Lameu and colleagues, who elucidated that a rich-club is highly related to network suppression and global neural synchronization of a network [48]. More detailed analyses of the cat’s cerebral network were performed in 2013 by de Reus and van den Heuvel, who defined rich-club members as the top 15 (23%) highest degree nodes with 11 regions based on a previous study [47] and four additional regions (i.e., suprasylvian fringe, dorsolateral division of the prefrontal cortex, anterior part of cingulate cortex, and anterior limbic cortex) [49]. Based on rich-club and non-rich-club nodes, existing connections in the cat network were classified into three categories: (1) rich-club connections only linking rich-club nodes, (2) feeder connections linking rich-club and non-rich-club nodes, and (3) local connections only linking non-rich-club nodes. Importantly, even with the lower connection density of rich-club connections (i.e., 14%, which was comparable to 48% and 38% for feeder and local connections, respectively), approximately 86% of the inter-modular communications were related to rich-club connections, which extended the role of rich-club brain regions to form a larger infrastructure for global and modular communication between different domains in the mammalian brain. The connections of the cat’s cerebral network were recently described according to two independent factors: relative cytoarchitectonic differentiation and spatial distance of brain regions, in which a linear combination of these two factors predicted the existence or absence of connections with >85% accuracy in the cat brain [50].

### 5.2 Rat and mouse

Using an open-access tract-tracing connectivity dataset on the rat [51], van den Heuvel and colleagues demonstrated that the white matter network of rats (with 67 nodes and 1396 connections) contained 14 rich-club members (~21%) with secondary motor, infralimbic, piriform, dorsal anterior cingulate, prelimbic, medial orbital, posterior agranular insular, temporal association, ectorhinal, perirhinal, lateral entorhinal, medial dorsal entorhinal, lateral amygdalar, and posterior basolateral amygdalar areas [52]. Similar to those in the cat, rich-club connections in the rat constituted 11% of total connections in the network, of which 75% were inter-modular, corresponding to significantly longer distances when compared to feeder and local connections. The association between large-scale network topology and molecular function in the mouse was reported in a transcriptional coupling study [53], in which the highest coupling observed in rich-club connections was driven by genes regulating oxidative synthesis and metabolism of ATP. This study suggested that the connections between brain hub regions are characterized by tightly coupled gene expression related to the regulation of oxidative metabolism. Specifically, approximately 46% of neuronal types comprised the rich-club in the rodent hippocampal neuronal network; indeed, this set had substantially tighter connections, termed *the richest of the rich* club [54].

### 5.3 Macaque

The cortical connectivity matrix (binarized with 242 nodes and 4090 connections) defined from 410 neural tracing studies of the macaque revealed rich-club regions in the prefrontal, paracingulate, anterior cingulate, parietal, and temporal cortices [55]. Consistent with those in other species, rich-club regions in the macaque have inter-modular connections rather than isolated subnetworks, and global information flow is mediated by these regions [56]. Moreover, microscale cortical neuronal complexity is associated with macroscale network topology (e.g., degree), particularly in rich-club regions [57], highlighting the importance of neuronal architecture within macroscopic brain networks. Furthermore, structural connectivity is positively associated with resting-state functional connectivity, in which rich-club regions exhibit the greatest functional stability over time depending on their structural topology [58]. More specifically, a small number of neurons in a network seem to be strongly interconnected with oscillatory synchrony to form a rich-club [59]. These neuronal regions may eventually lead to more complex network dynamics in terms of brain function [60], in which connectivity strength could enhance neuronal synchronization, particularly between rich-club regions [61].

### 5.4 Rich-club implications in mammals

**Fig. 3:**
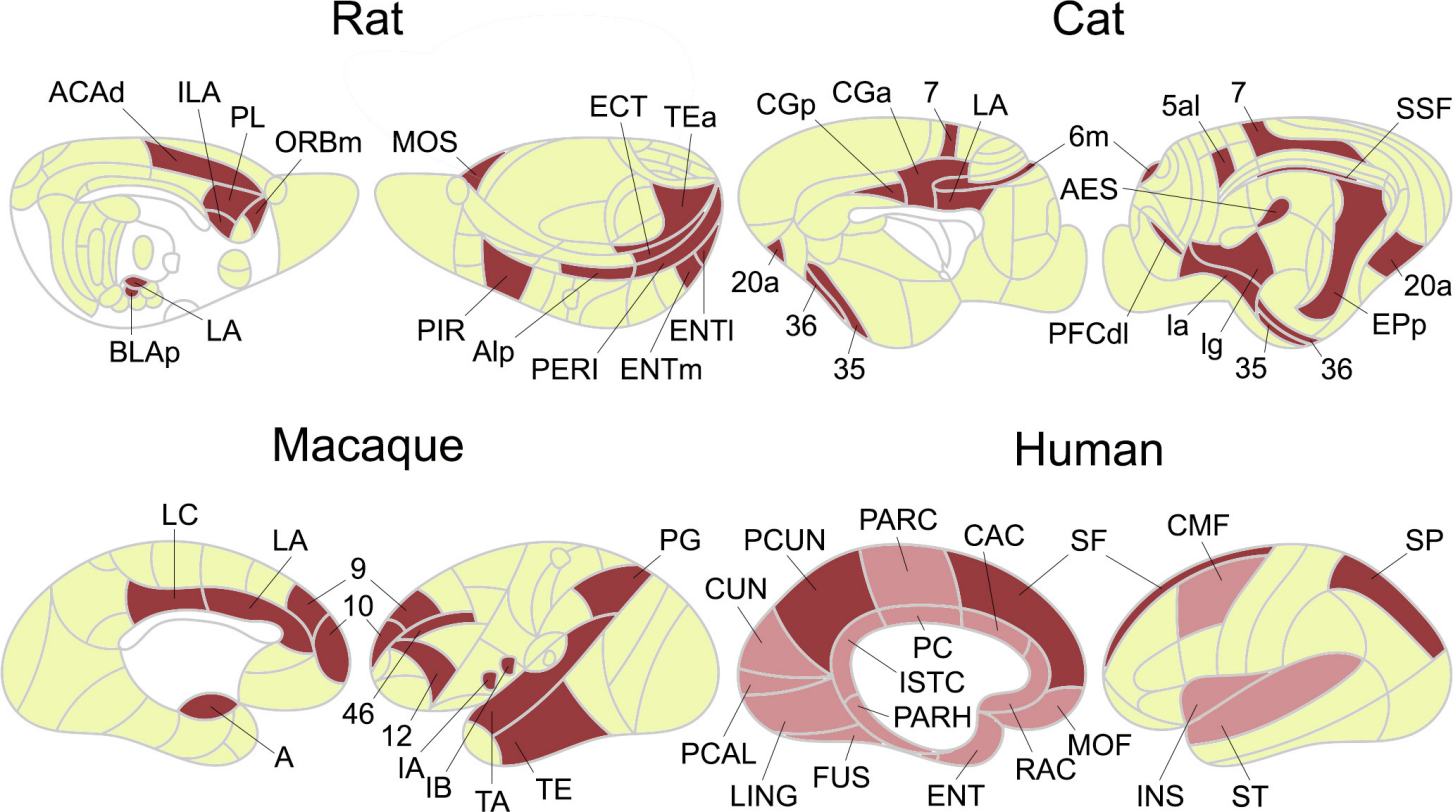
Rich-club regions in different species. Brown colors represent the cortical rich-club regions in the rat [52], cat [49], macaque [57], and human [20]. The rat connectivity data were obtained from the open-access BAMS-II database (https://bams1.org) [51] and parcellated into 67 regions and 1397 directed axonal projections. The cat cortex was parcellated into 65 regions and 1139 axonal projections as reported by Scannell and colleagues [44], resulting in a binarized connectivity matrix. The macaque axonal projections are based on the open-source CoCoMac database (http://cocomac.g-node.org/main/index.php) with 39 × 39 unweighted directed connectivity matrices. The human brain is parcellated using Freesurfer (http://freesurfer.net) into 34 (low resolution) and 1170 (high resolution) cortical regions, and the corresponding rich-club regions are depicted in dark and light brown colors. (For interpretation of the references to colour in this figure legend, the reader is referred to the web version of this article.)

While rich-club neurons in *C. elegans* consist primarily of command interneurons, macroscopic rich-club regions are consistently detected in mammals such as the rat, cat, and macaque; i.e., a set of highly connected and central brain regions forming a densely connected rich-club. In mammals, rich-club regions are more highly spatially distributed across the whole brain to facilitate inter-modular communications rather than intra-modular connections. Further, mammalian rich-club regions exhibit greater connection strength among themselves than feeder and local connections (Fig. 3). While caution should be exercised in cross-species comparisons [62], the considerable overlap in rich-club organization across mammals suggests the existence of “common” biological substrates across mammalian species that may contribute to the integration of neural information resulting in optimal behavioral functioning. Of note, the detailed aspects of rich-club distribution may be related to the characteristics of a species. For example, the rich-club members in the macaque include more prefrontal regions than those in the rat and cat (Fig. 3), which may be related to the higher-order behavioral functions in this species.

## 6 Rich-club of humans

Rich-club organization in the human brain was first examined using DTI, in which 12 bilateral rich-club regions from 82 cortical and subcortical parcellations were consistently detected in the superior parietal lobule, precuneus, superior frontal cortex, putamen, hippocampus, and thalamus [20], [63]. Consistent with those in the macaque, cat, and rat, all brain regions had at least one link connected to rich-club members, suggesting a key role in global information communication in distant regions via crucial areas in the brain. Simulations of the extent to which the brain network is damaged from an “attack,” defined by an arbitrary decrease in connection strength, to a certain set of connections (e.g., (1) “targeted attack” only to rich-club connections, (2) “hub attack” to feeder connections, and (3) “random attack”) have revealed that measures of network efficiency are decreased, particularly in rich-club attacks. These findings underscore a potential framework for connectivity disturbances linked to neuropathology. Rich-club regions constitute approximately 69% of all connection paths with 40% of the total communication cost (defined by the number of streamlines based on physical length between two regions), implying the high cost and capacity of these regions for global brain communication [22]. Moreover, structural rich-club regions are strongly associated with resting-state functional networks and regional volume, metabolic energy use, maturation, temporal variability, and structural–functional associations [21], [64], in which the involvement of each functional network of the rich-club varies from a minimum of 3–9% (e.g., extrastriate visual, motor, sensory, and auditory networks) to a maximum of 22% (e.g., default-mode network) [65]. These findings highlight the relevance of centrally collective brain structures in the flexible and reconfigurative nature of brain community organization for integrative cognitive function [65], [66], [67]. Key findings from human rich-club neuroimaging studies are summarized in Table 2.

**Table 2:** Summary of outcomes from human rich-club neuroimaging studies.

Refs.	Sample size	Age (mean ± SD years)	Modality	Parcellation: Number of network nodes	Connectivity	Rich-club findings
van den Heuvel et al. [20]	21	29.95 ± 8.3	DTI	82 and 1170 for low and high resolution	Deterministic fiber tractography. SC is binary or weighted (*N_f_*, *N_f_* normalized by regional volumes, and *N_f_* × FA).	First RC analysis in the human brain. RC exists in the bilateral superior parietal, precuneus, superior frontal cortex, putamen, hippocampus, and thalamus.
Kocher et al. [63]	43	37.1 ± 11.7	DTI	82	*N_f_* corrected by their length and regional volume.	RC nodes are symmetrically distributed across all brain regions. Marked anatomical consistency of RC regions exists.
van den Heuvel et al. [22]	80	Set 1: 28.6 ± 7.9, Set 2: 27.0 ± 6.9	DTI	1170	*N_f_*	Network cost (defined as *N_f_* × path length) of RC connections was high (~40% of the total cost). About 69% of connection paths pass through RC.
van den Heuvel et al. [64]	77	28.2 ± 8.0	DTI and rs-fMRI	DTI was processed as in [22]. RSNs were extracted with ICA to rs-fMRI.		RC nodes are present in all RSNs and coincide in regions with multiple RSNs. Inter-RSN connections are involved in RC.
Collin et al. [21]	42	29.0 ± 8.0	DTI, MTI, and rs-fMRI	68 cortical regions	SC = FA, MD, TD, and PD. FC = CC	RC regions and connections have high levels of volume, white matter organization, metabolic energy usage, long maturational trajectories, more variable regional time-series, and more inter-regional functional couplings.
Crossley et al. [65]	Meta-analysis (>1600 studies)		rs-fMRI	638 brain regions	Coactivation matrix	Functional RC is located in the parietal and prefrontal cortices. It is connected over long distances and coactivated by multiple tasks.
Baggio et al. [66]	30	66.2 ± 10.4	DTI	68	*N_f_*	RC connectivity is associated with general cognitive performance.
Miŝic, et al. [67]	156 subjects in HCP		DTI and rs-fMRI	114	FA and *N_f_*	Structural RC connections are more involved in network-level SC–FC associations. RC regions are disproportionately involved in network-wide communication.
Ball et al. [24]	46	27^+1^ (24^+4^–34^+5^) gestational weeks	DTI	~500 regions, probabilistic fiber tractography, and binarized SC matrix.	1 for connected and 0 for disconnected regions	RC is present by 30-week gestation. Connections between RC and the rest of the brain proliferate until the time of normal birth. RC organization remains intact following premature birth, but both cortical–subcortical connectivity and short-distance corticocortical connections are disrupted.
Kim et al. [69]	147	8.12 ± 1.35	DTI	90 cortical and subcortical regions	FA of interconnecting fiber tracts	Connectivity strength is higher: (1) among RC nodes in children with longer gestation, and (2) in RC nodes compared to the feeder and local connections.
Grayson et al. [68]	14 adults and 15 children	Adults: 24–35, children: 7–11	DTI and rs-fMRI	219 cortical regions	*N_f_*	Both adults and children have similar structural RC organization, but the adults have greater functional RC organization.
Perry et al. [70]	115	76–94	DTI	512 cortical and subcortical regions	*N_f_* corrected by their length	RC organization is consistent in both elderly and young adults.
Zhao et al. [71]	113	38.2 ± 21.4, 9–85	DTI	1024 cortical and subcortical regions	*N_f_*	Structural RC connectivity has an inverted U-shaped trajectory across the lifespan. RC regions are distributed in the medial frontal, parietal, and occipital cortices in children and young adults, but RC connections in the frontal regions are reduced in older age.
Cao et al. [72]	126	36.8 ± 21.2, 7–85	rs-fMRI	1024 cortical and subcortical regions	CC	Functional RC connectivity has an inverted U-shaped lifespan trajectory.
Zhao et al. [73]	77	25.0–41.4 weeks	DTI	58 cortical regions	*N_f_* × FA	Efficiency of RC networks is increased more rapidly than that of non-RC networks in term-born brain networks.
Baker et al. [74]	31	16.58 ± 0.54	DTI	80 cortical and subcortical regions	*N_f_* and FA	RC connectivity between subcortical regions decreased over time. Frontal–subcortical and frontal–parietal hub–hub connectivity is increased over time.
Kim et al. [75]	99	7.80 ± 1.22	DTI	90 cortical and subcortical regions	FA of interconnecting fiber tracts	RC connectivity has a positive association with children's intelligence score. Children's ability for visuo-motor spatial reasoning showed significant correlations with RC.
van den Heuvel et al. [76]	27	30–42 gestational weeks	DTI and rs-fMRI	56 cortical regions	SC = FA, MD, TD, and PD. FC = CC	Neonatal RC regions are located in the left superior frontal, left lateral front-orbital, left precentral, left postcentral, left and right superior parietal, left and right cingular, left and right angular, and left and right fusiform areas.
Kim et al. [77]	49	6.2–9.4	DTI	1015 cortical regions	FA	Positive associations between prenatal maternal cortisol level and network cost are found for RC connections of girls only at 31 gestational weeks.
Scheinost et al. [78]	12 preterm and 25 term neonates	Preterm: 27 ± 2.2,Term: 40 ± 1 weeks	rs-fMRI	95 cortical and subcortical regions	CC	Both preterm and term neonates have RC organization, but preterm neonates have reduced RC connectivity.
Fischi-Gomez et al. [79]	51	26.7–32.5 gestational weeks	DTI	82	*N_f_* corrected by their length and regional volume	No RC differences between extreme prematurity (EP) and intrauterine growth restriction (IUGR) groups. RC organization after premature birth is maintained even at school age.
Karolis et al. [80]	51 preterm and 60 controls	38–42 gestational weeks	DTI	82 cortical and subcortical regions	*N_f_*	Very preterm brains exhibit stronger RC architecture.

*DTI*, diffusion-tensor imaging; *FA*, fractional anisotropy; *CC*, correlation coefficient; *SC*, structural connectivity; *FC*, functional connectivity; *RC*, rich-club; *N_f_*, the number of fiber tracts between two regions; *rs-fMRI*, resting-state fMRI; *RSN*, resting-state networks; *ICA*; independent-component analysis; *MTI*, magnetic transfer imaging; *HCP*, human connectome project; *MD*, mean diffusivity; *TD*, transverse diffusion; *PD*, parallel diffusion.

### 6.1 Application to brain development

Rich-club organization is established prior to the time of birth [24]. It develops throughout childhood, adolescence, and adulthood [68], [69], [70], [71]; and is sustained throughout the lifespan [72]. Specifically, the connection density between rich-club regions and the rest of the cortex increases during the third trimester [24] in neonates [73], and even in late adolescence [74]. The intelligence quotient of typically developing children exhibits a stronger positive association with rich-club connectivity than with feeder and local connectivity [75]. Although developmental trajectories in the structural and functional rich-clubs are similar in neonates and adults [76], adults exhibit greater functional rich-club organization compared to that in the younger population [68]. The cost of rich-club connections is strongly associated with prenatal maternal cortisol levels, a measure of maternal stress during pregnancy; this effect is unique to women, suggesting sex-specific rich-club contributions during development [77]. The development of term and preterm children using rich-club analyses is an active research area. Preterm neonates were reported to have reduced connectivity of rich-club regions [78], and children with longer gestation exhibit more efficient structural networks with higher rich-club connectivity when compared to children with shorter gestation [69]. In contrast, rich-club characteristics associated with preterm births have been reported to be maintained in school-age children [79] or to be stronger in adults [80]. These findings emphasize that the overall topological characteristics in the brains of children are associated with longer gestation, and shorter gestation does not significantly impact rich-club organization particularly in children (Table 2). In summary, the structural and functional rich-club framework provides a method to map the age-dependent patterns of brain development and to answer several key questions: (1) What are the critical roles of “core” brain regions in brain development? (2) Can improved development of rich-club organization lead to improved brain function? (3) Are peripheral connections merely supplementary to brain functions when compared to rich-club connections? Empirical studies have demonstrated that brain network evolution is particularly centered with rich-club regions in the developing brain. However, critical challenges related to the network development should be addressed in future studies, which include: (1) the precise mapping of developmental stages in both structural and functional rich-clubs, (2) the neural substrates of abnormal rich-club development, and (3) the predominance of rich-club versus non-rich-club regions in network development.

### 6.2 Clinical findings: Psychiatric disorders

Both structural and functional brain networks exhibit rich-club organization in schizophrenia [23], [81], [82], [83], suggesting that the effects of altered brain connectivity are more concentrated in rich-club connections than in feeder or local connections in patients with schizophrenia. The connection density of rich-club regions derived from DTI and white matter tractography is often observed to be reduced in patients with schizophrenia, while rich-club density is intermediate in unaffected siblings relative to that in patients and healthy controls, and is lower in their offspring than in healthy controls [25], [84], [85]. These findings have been replicated in subjects with high clinical risk for psychosis [86]. Furthermore, decreased rich-club connection density is associated with lower levels of global communication capacity (i.e., network efficiency), resulting in a stronger association between structural and resting-state functional connectivity in patients [23]. Importantly, the increased structural–functional association in patients is interpreted as more stringent and less dynamic brain function from the illness that is more directly associated with the underlying structural connectivity. Additionally, higher connectivity strength of rich-club connections is associated with positive changes in general functioning over time in schizophrenia [87], [88]. Rich-club connections among rich-club nodes are lower in major depressive disorder (MDD) and late-life depression patients than in healthy controls, in which higher rich-club connectivity is associated with lower symptom severity score (i.e., Hamilton Depression Rating Scale) [89], [90]. However, the remission of MDD patients is more strongly associated with feeder-local subnetworks than with rich-club connections [91], suggesting that compensatory effects from treatment may be more distinct in non-core brain regions in patients with this psychiatric disorder. Additionally, the development of age-related rich-club organization has been reported in typically developing adolescents but not in patients with autism spectrum disorder (ASD) [92]. Ball and colleagues reported that the rich-club connections in ASD patients exhibited an inverted U-shaped association with age [93], which is similar to that in healthy controls [71]. While the phenotypes of impaired neurodevelopmental disorders such as attention deficit hyperactivity disorder (ADHD) and ASD overlap substantially in terms of clinical comorbidity, ADHD and ASD children exhibit distinct patterns of rich-club and non-rich-club connections [94]. Global network efficiency has been reported to be decreased in bipolar disorder (BD) patients, but no significant differences have been noted in the strength of brain hub connections with rich-club regions [95], [96], [97], suggesting that aberrant network organization may not be specific to the central core system of BD. However, more recent studies have indicated that BD patients possess decreased rich-club and feeder connectivity density [98] and increased rich-club connectivity [99].

### 6.3 Clinical findings: Neurological disorders

Rich-club organization is more predominant in patients with Alzheimer’s disease (AD), and a recent study suggested that rich-club connectivity (as measured by the fiber density interconnecting two regions) is decreased in the early-onset AD [100]. However, low-degree regions, and not rich-club regions, have been found to be more strongly associated with network disruption in AD patients [101], [102], [103]. This suggests that peripheral connections may be more vulnerable and contribute to cognitive decline in this neurodegenerative disease. Moreover, patients with generalized tonic-clonic seizures have reduced rich-club connectivity, which is associated with longer durations of illness and seizure frequencies [104]. Structural connectivity of rich-club regions is decreased in patients with multiple sclerosis, in whom decreased rich-club connectivity is associated with mobility, hand function, information processing speed [105], and cognitive impairments [106]. In patients with traumatic brain injury (TBI), the strength of local connections is increased, but rich-club connectivity is decreased [107]. These results have been replicated in cognitively impaired and nonimpaired active professional fighters [108]. These findings suggest that peripheral subnetworks may compensate for biologically high-cost rich-club subnetworks after TBI. Finally, decreased structural connectivity has been observed in rich-club regions in patients with cerebral small vessel disease, which was positively associated with psychomotor speed and executive function [109]. However, connectivity disruption in rich-club regions did not have specific effects over time, as observed in a longitudinal study [110].

### 6.4 Rich-club implications for brain disorders

Highly interconnected network hub regions often form rich-clubs. A coactivation network *meta*-analysis using functional neuroimaging data revealed that topological characteristics such as network module, small-worldness, and rich-club are often consistent across psychiatric and neurological brain disorders [111]. However, pathological lesions are likely to be found in hub regions rather than peripheral regions, whereby rich-club regions have lesions twice as often as peripheral network nodes. These findings suggest that: (1) brain regions do not function equally in brain network architecture, (2) brain disorders are more strongly associated with damage to central brain regions such as rich-clubs, and (3) the disruption of network rich-clubs may be common across various brain diseases. Of note, the relationship between a specific brain dysfunction and network rich-club regions is dependent on the “location” of lesions. For example, while schizophrenia and AD share hub-specific distributions of lesions, regions more strongly implicated in each disease are located in the frontal and cingulate regions for schizophrenia, and in the medial temporal and parietal regions for AD.

## 7 Summary and outlook

The functional roles of biological network elements vary according to their anatomical locations. This differentiated functional organization of the central nervous system has often been associated with specific functions of the network system such as sensorimotor function, mental activities, and behaviors [112]. The concept of “functional specialization” is supported by various neurophysiological, anatomical, and noninvasive neuroimaging findings, and has formed a theoretical neural substrate underlying cognition. However, the complex nature of human cognition prompts the following question: How do functionally specialized units communicate with each other optimally? The conceptual framework used by researchers to understand neural systems such as the brain emerged from the idea that individual neural elements are functionally integrated and orchestrate higher-order brain activities such as sensory recognition, emotion, language processing, and social cognition in a coordinated manner [113]. The organization of functionally segregated and anatomically integrated biological systems has been investigated from the perspective of complex network theory [114]. Network science or graph theory has revealed a structural basis for the dynamic functional interactions emerging from a diverse set of neural elements and defined how structural topology gives rise to modular brain function ranging from *C. elegans* to mammals and humans. A key organizational feature is the existence of crucial elements that attribute functional specialization to neural networks, known as *hubs.* Hub elements have been found to produce efficient neural information flow at the expense of neural cost [115]. They have more connections (i.e., higher degree) or higher levels of connectivity, particularly for long-distance connections exhibiting the “rich” aspects of hub elements, forming the “rich-club” [20], [22].

Several important issues arise from the current rich-club perspective and network analysis. It is necessary to develop optimal computational and mathematical frameworks for each data modality in each neuronal system in terms of network construction, statistics, and their interpretations [116]. First, defining network nodes and edges is crucial for modeling neural systems [117] and depends strongly on the research domain (e.g., anatomical and functional networks acquired from neuronal, physiological, and neuroimaging datasets). Regarding macroscale brain networks (i.e., brain regions), parcellation techniques have been used to subdivide the whole brain into anatomically distinct areas resulting in segregated nodes of the brain network. Such parcellations include predefined anatomical segregations (e.g., Brodmann areas, Desikan-Killiany atlas [118], and anatomical automatic labeling map [119]), atlas-independent random cortical segmentation [24], and data-driven clusters, which are often derived from functional correspondence [120], [121], [122], [123] with individualized state-specific parcellations [124], [125] (refer to the study by Arslan and colleagues [126] for a comparison of each parcellation scheme). While the predefined parcellation templates integrate network properties across each individual leading to unbiased group comparisons, it should be noted that the size and distribution of each parcel modulate quantitative topological properties including the rich-club organization of a brain network [127], [128]. The significance of network edges is also considered important. Anatomical neuroimaging techniques represent diverse definitions of structural connectivity between two brain regions (e.g., the number of streamlines generated from fiber tractography, tract-based diffusion characteristics such as fractional anisotropy and mean diffusivity, cortical thickness, and the amount of myelination in white matter) [20], [129], [130], while functional edges are designated by functional similarity such as Pearson correlation coefficient, covariance, coherence, and mutual information measured with fMRI, EEG, and MEG recordings. In addition to the definitions of structural and functional connectivity, functional networks are intrinsically more dynamic than structural networks and act as determinants of brain function and dysfunction that are constrained by brain structure [131]. Furthermore, weighted structural and functional connectivity measures are often noisy due to physiological and methodological limitations. Binarization or thresholding techniques may be applicable to enhance the contrast between relevant and irrelevant connectivity values [132]. However, binarization or thresholding is highly dependent on whether the connectivity is absolute (retaining values over a threshold) or proportional (retaining a fixed percentage of values) and may have a greater impact on certain global network measures particularly derived from functional connectivity [133]. In addition, a general consensus on the definition of network threshold is lacking, and researchers use empirical values to determine thresholds. These binarization or thresholding techniques may diminish or exaggerate connectivity values below or above the threshold, leading to under- or over-estimated network characteristics, respectively. It is challenging to determine whether individual or group-wise variations in rich-club regions are indicative of methodological limitations resulting from the aforementioned computational and technical challenges, or whether they truly reflect additional biological information in terms of inter- and intra-individual variability. For instance, rich-club regions identified in an initial report [20] consist of the superior parietal and frontal cortex, precuneus, putamen, hippocampus, and thalamus. However, more recent studies have identified the insula as another rich-club region [21], [24], [74], [77]. This may arise from the arbitrary threshold for rich-club detection based on statistical rich-club coefficients *ϕ*(*k*) (e.g., top 10% [24], [77], 12% [21], and 18% [74]). Since the number of connections across nodes in the brain network often increases gradually and not distinctively, a more liberal or conservative definition of the rich-club may affect subsequent analyses. Thus, although the rich-club organization exists regardless of definitions of brain parcellation and connectivity, these may affect qualitative characteristics of the rich-club, reflecting distinct aspects of rich-club regions in the brain network. In this regard, a robust and consistent rich-club characterization remains unsolved in brain network science.

Rich-club analysis and whole brain anatomical network analyses are often based on neuroimaging techniques such as DTI and fiber tractography. While technological advancements in these techniques are rapidly increasing, the intrinsic nature of neuroimaging remains an open question. First, fiber tractography is a deterministic approach which often provides one-to-one connections from a seed point that may miss crossing, splitting, and/or branching tracts. Although probabilistic algorithms have been applied as an alternative to resolve this issue, other false-positive connections may be detected. This approach may not comprehensively assess rich-club detection and connections because fiber tracts related to the rich-club are relatively insensitive to false-positive and false-negative tracts. However, feeder and local connections are highly dependent on the quality of fiber tractography and may have a larger impact on peripheral associations to rich-club regions. The development of better qualified tractography algorithms is required. Second, similar to conventional graph theoretical analysis, the rich-club organization is relatively dependent on the definition of structural connectivity derived from DTI. For example, the number of streamlined fiber tracts is commonly used for the detection of the rich-club and other network measures. In addition to variations in streamlines related to parameter adjustment during fiber-tracking, investigating the association between the connections with neuronal tracing as true connectivity and the number of streamlines or other variations (e.g., scalar measures along fiber tracts such as fractional anisotropy) is recommended. Third, the structural and functional rich-club organization extracted from neuroimaging is strongly associated with genetic variation [53], [134], structural–functional coupling [23], [84], and metabolism [135]. Therefore, higher cost and central roles of rich-club regions may be established based on the high metabolic energy consumption in these core regions and coupled with certain patterns of gene expression related to metabolism. This may lead to higher levels of functional and structural connections with larger gray matter volume and improved white matter microstructure, thereby establishing a closer relationship between brain structure and function. To establish the consequences of rich-club formation in the brain, various modalities including genetic, metabolic, microbiological measurements, and neuroimaging are required to elucidate the convergent implications of brain rich-club organization. Fourth, graph theoretical brain network analysis, which enables the investigation of rich-club organization, is dependent on the scales-of-interest at multiple levels for a given network (i.e., global, modular, or local network organization). Thus, detection of the rich-club architecture at the level of individual regions is often associated with modular or global brain network properties. In addition to the global communication efficiency derived from rich-club architecture (i.e., role as connector hubs), rich-club nodes often form subnetwork communities or modules with a relatively sparsely connected set of nodes (i.e., peripheral nodes). The implications of each rich-club node (as in psychiatric and neurological disorders) may exert its influence on the participating module within the network rather than on the individual node itself (i.e., role as provincial hubs). Therefore, analyses at the level of intermediate-networks (i.e., modules) and individual rich-club nodes are crucial to extend the utility of these central and rich network units. Of importance, detection of network modules or communities is dependent on the applied optimization algorithm, often resulting in non-unique network subdivisions, which may restrict the unbiased interpretation of consistent modular structures related to specific rich-club nodes. Deriving strategies for optimal modular detection techniques will be crucial to understand the resilience and vulnerability of rich-club nodes at the level of higher-order network structures. Fifth, the rich-club comprises a set of core brain regions, which is relatively invulnerable to external attacks (i.e., brain disease) because the brain has several neural resources (i.e., high cost). This suggests that rich-club abnormalities are more easily detected when the attack exceeds the resistance and tolerance to external insults (i.e., critical point). Therefore, beyond conventional graph theoretical measures, rich-club investigations of the brain network should pay more attention to determining thresholds at which external attacks exceed a critical point (i.e., occurrence of a certain disease).

In this review, we have described how rich-club organization has been applied to neural systems to reveal neuroanatomical correlates with brain development and disorders. Network neuroscience investigates brain structure, function, behavior, and cognition. Considering the weaker statistical power of typical clinical studies, systematically collected large-scale datasets from multicenter and multimodal data collection (e.g., Human Connectome Project [136], [137], [138]) will be essential to comprehensively assess the phenomenon of rich-club organization and understand the neural architecture underlying brain development and disorders. Making sense of these brain network datasets presents an exciting challenge of bridging the gap between topological findings related to core brain regions and the biological significance of computational interpretations.

## Declaration of Competing Interest

The authors declare that they have no known competing financial interests or personal relationships that could have appeared to influence the work reported in this paper.

## Footnotes

1 Network rich-club can be computed with BCT (https://sites.google.com/site/bctnet/) in MATLAB, braingraph (https://github.com/cwatson/brainGraph) in R, or NetworkX (https://networkx.github.io/documentation/networkx-1.9.1/index.html) and bctpy (https://pypi.org/project/bctpy/) in Python.
